# Pharmacogenomics driven decision support prototype with machine learning: A framework for improving patient care

**DOI:** 10.3389/fdata.2022.1059088

**Published:** 2022-11-15

**Authors:** Farah Kidwai-Khan, Christopher T. Rentsch, Rebecca Pulk, Charles Alcorn, Cynthia A. Brandt, Amy C. Justice

**Affiliations:** ^1^VA Connecticut Healthcare System, West Haven, CT, United States; ^2^Yale School of Medicine, New Haven, CT, United States; ^3^Faculty of Epidemiology and Population Health, London School of Hygiene and Tropical Medicine, London, United Kingdom; ^4^Yale New Haven Health Pharmacy, New Haven, CT, United States; ^5^Yale School of Public Health, New Haven, CT, United States

**Keywords:** pharmacogenomics, machine learning, clinical decision support, data framework, prototype

## Abstract

**Introduction:**

A growing number of healthcare providers make complex treatment decisions guided by electronic health record (EHR) software interfaces. Many interfaces integrate multiple sources of data (e.g., labs, pharmacy, diagnoses) successfully, though relatively few have incorporated genetic data.

**Method:**

This study utilizes informatics methods with predictive modeling to create and validate algorithms to enable informed pharmacogenomic decision-making at the point of care in near real-time. The proposed framework integrates EHR and genetic data relevant to the patient's current medications including decision support mechanisms based on predictive modeling. We created a prototype with EHR and linked genetic data from the Department of Veterans Affairs (VA), the largest integrated healthcare system in the US. The EHR data included diagnoses, medication fills, and outpatient clinic visits for 2,600 people with HIV and matched uninfected controls linked to prototypic genetic data (variations in single or multiple positions in the DNA sequence). We then mapped the medications that patients were prescribed to medications defined in the drug-gene interaction mapping of the Clinical Pharmacogenomics Implementation Consortium's (CPIC) level A (i.e., sufficient evidence for at least one prescribing action) guidelines that predict adverse events. CPIC is a National Institute of Health funded group of experts who develop evidence based pharmacogenomic guidelines. Preventable adverse events (PAE) can be defined as a harmful outcome from an intervention that could have been prevented. For this study, we focused on potential PAEs resulting from a medication-gene interaction.

**Results:**

The final model showed AUC scores of 0.972 with an F1 score of 0.97 with genetic data as compared to 0.766 and 0.73 respectively, without genetic data integration.

**Discussion:**

Over 98% of people in the cohort were on at least one medication with CPIC level a guideline in their lifetime. We compared predictive power of machine learning models to detect a PAE between five modeling methods: Random Forest, Support Vector Machine (SVM), Extreme Gradient Boosting (XGBoost), K Nearest neighbors (KNN), and Decision Tree. We found that XGBoost performed best for the prototype when genetic data was added to the framework and improved prediction of PAE. We compared area under the curve (AUC) between the models in the testing dataset.

## Introduction

The United States Department of Veterans Affairs (VA) was one of the pioneers in adapting the electronic health records (EHR) in the mid-1990s, resulting in standardized data available for research from 1999 onwards. The Veterans Health Administration (VHA) is the branch of the VA that provides healthcare to Veterans. It has grown from 54 hospitals in 1930–1600 healthcare facilities, including 144 medical centers and 1,232 outpatient sites of care. Patient care is dependent on the framework within which clinicians make treatment decisions and provide care (Hicks et al., 2016). The framework includes all systems starting from patient enrollment, diagnosis, treatment, medications, hospital stay, discharge, follow-up, and long-term disease management. Clinical decisions are dependent on this data; therefore, it would be enabling for the clinician if this data is transformed from medical information to a knowledge source that leads to precision care based on available guidelines.

The inclusion of genetic data within the EHR is infrequent, highly variable and not yet standardized. Routine clinical care treatment decisions do not factor genetic data although the success rate of medications and procedures may vary depending on the genetic profile of the patient. Literature shows that in the U.S. alone, approximately 770,000 injuries or deaths occur every year due to an inappropriately prescribed medication (Brownlee and Garber, 2019; Matsuyama et al., 2021) with estimated expenses between “$1.56 and $5.6 billion annually” (Slight et al., 2018). The Center for Disease Control (CDC) reported that between 2011 and 2014 more than 66% of people over 65 took three or more prescription medications (Carstens et al., 2009; Veteran Affairs, 2021). An aging population with multiple medications can lead to potentially inappropriate prescribing (Hoel et al., 2021) of medications (PIMs) (Bradley et al., 2017; Burkhardt et al., 2020). This research is an attempt to address some of the systemic issues that contribute to medication related clinical issues. Systems are fragmented (Caudle et al., 2014), and the availability of resources to enable the right decision-making is piece meal (Hicks et al., 2016; Matsuyama et al., 2021). Guidelines may exist but are not integrated into a tightly coupled well-managed data framework (Al Kawam et al., 2018; Kidwai-Khan, 2022).

People with HIV frequently take multiple medications and are susceptible to chronic diseases or comorbidities specifically hepatitis C, pain, diabetes and cardiovascular disease. Intake of multiple medications concurrently increase the risk of adverse drug events (Young et al., 2021). These factors make the decision-making process more complicated. In the VA the large pool of genetic data that is available provides a rich resource for studying an integrated health record and its impact on decision making. The proposed framework integrates individual systems that can effectively interact to facilitate meaningful interpretation and decision making. The study used predictive modeling and unique data management methods to create and validate algorithms to enable informed decision-making for clinicians. Machine learning was explored as a method to perform predictive outcomes. The goal was to assess if an integrated data framework combining EHR data with relevant elements of genetic data and decision support based on predictive modeling improve prediction of PAE.

## Methods

### Data source

The VA has more than 20 years of EHR data available in a corporate data warehouse (CDW). This data is kept live and up to date to reflect the EHR. The Veterans Aging Cohort Study Biomarker Cohort (VACS-BC) is a cohort of 2,656 patients (1,721 HIV-infected and 935 matched uninfected controls) with genetic data based on blood specimens provided between 2005 and 2007 or consented for DNA analyses. Subjects in the sample were between 41 and 64 years of age (>80%) at the time of enrollment. Most were men (95% male) of African American ancestry (68%).

The sample used for this study included 2,471 patients after excluding those had died before 2018. For these patients, we extracted their EHR data from 2018 to 2021, including information on diagnoses, labs, and medications. Medication data include medication generic name, date dispensed to patient, dosage, quantity, refills, prescriber, pharmacy and total days on medication.

Programming algorithms are run on this to create a list of medications for each person over time and stratified by past, current, active or inactive. The algorithms also create a rollup regimen for each patient that creates start and stop dates from when a medication was started and when it was stopped. This is linkable to diagnoses and progress notes to develop a clinical story for a given patient.

We also extracted genetic data, which is stored as a series of single nucleotide polymorphism (SNPs pronounced snips). These are positions on a gene where some individuals have one nucleotide, e.g., a G, whereas others could have a C. The information in DNA is stored as a linear code made of four chemical bases: adenine (A), guanine (G), cytosine (C), and thymine (T). SNPs are single base changes, like a single letter variation (like “analy*z*e” vs. “analy*s*e”). These are variations, not mutations. We also extract other data elements such as PBMCs (peripheral blood mononuclear cell: blood cell with a round nucleus), serum plasma, RBCs (red blood cells), and DNA (deoxyribonucleic acid) in addition to self-reported survey data available through VACS.

### Data cleaning, integration, and preprocessing

As data is in a data warehousing format, it needs to be extracted, processed and cleaned to be available in an analyzable format. For this study, we sourced data from the CDW, and all programming for data management was done using structured query language (SQL) and statistical analysis system (SAS). Data extraction was done with extract, transform and load (ETL) processes. SQL Server data tool was utilized to create routines in the.NET framework common language runtime environment. Data from different EHR domains such as demographics, diagnosis, pharmacy, and visits were all integrated into one SQL Server database. Interoperability issues were resolved by creating algorithms in SQL that could read data from a generic flat file format and converted to a SQL table. Different formats were brought into SQL as a single source. Variables such as age, sex, race date of diagnosis, comorbidities, medication name, drug class, dose, quantity and prototyped genetic data with gene and allele variant were transformed to created clean versions from raw tables. Composite variables were created that rolled up medication intake by date were matched to genetically relevant data and transformed into analytically meaningful datasets. Table 1 shows the variables for the dataset.

**Table 1:** Finalized variables in dataset after cleaning.

**Name**	**Independent/Dependent**	**Description**	**Values**
UID		Unique ID assigned for each patient	Randomly assigned unique Integer value
Sex	Independent	Sex of patient	1 = Male, 2 = Female
HIV	Independent	If person has HIV	1 = Yes, 0 = No
Anyca	Independent	If person has any cancer	1 = Yes, 0 = No
Diabetes	Independent	If person has diabetes	1 = Yes, 0 = No
Cardiovascular disease	Independent	If person has cardiovascular disease	1 = Yes, 0 = No
Treatment	Independent	Prescribed treatment	Treatment name
GeneAllele	Independent	If person is positive for indicated gene	1 = Positive, 0 = Negative
PAE	Dependent	Can the gene/medication values create a PAE	0 = No, 1 = Yes

PAE, Preventable Adverse Event.

**Figure 1:**
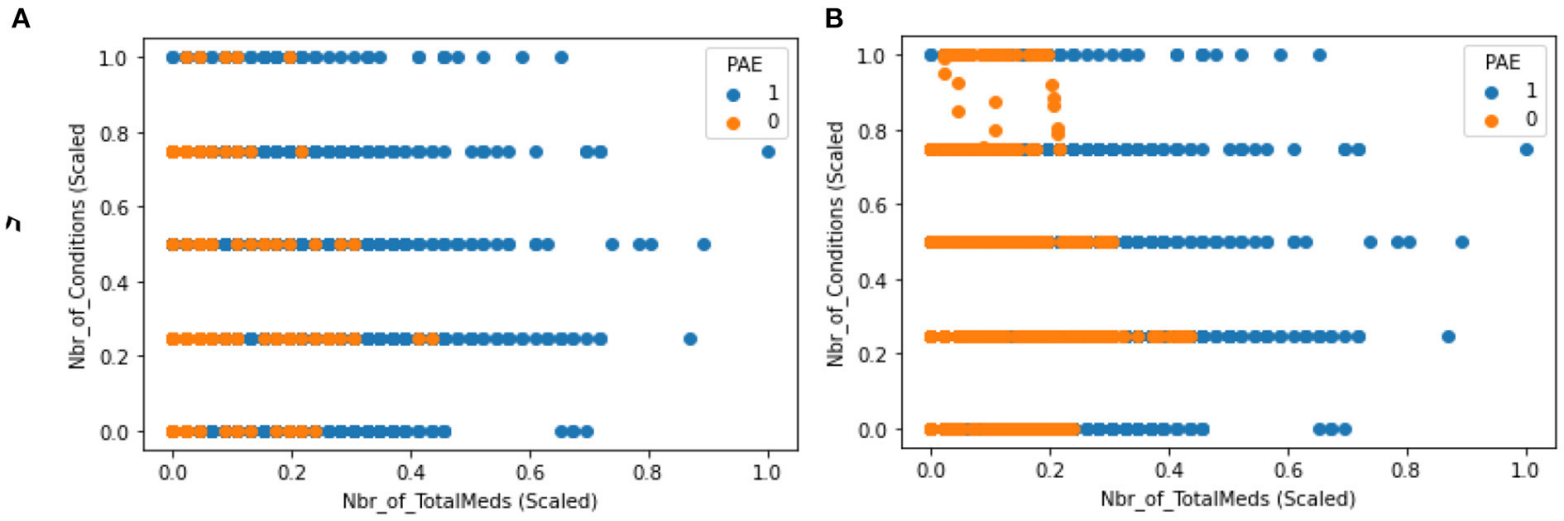
**(A)** Distribution before resampling **(B)** distribution after resampling.

Feature scaling was performed to transform feature values into a similar range. This is required by distance-based methods such as KNN and SVM (Choi, 2021). Some features in this data, such as total days on a medication, had a wide range (min: 30, max: 3517) compared to others (min: 0, max: 1). Given that distributions of these features are not normal, the “Min-Max” scaler was utilized to transform range to 0 and 1 (0, 1). This type of data is called imbalanced and is harmful for performance for machine learning models. This happens as the training model spends most of its time on the majority observations not learning enough from minority observations. To address this issue, Synthetic Minority Oversampling Technique (SMOTE) was utilized to oversampling the minority observations. SMOTE was only applied to the training dataset. The scatter plots in Figure 1 compare distribution of datapoints before and after resampling for applying SMOTE.

### Predictive analytics

**Figure 2:**
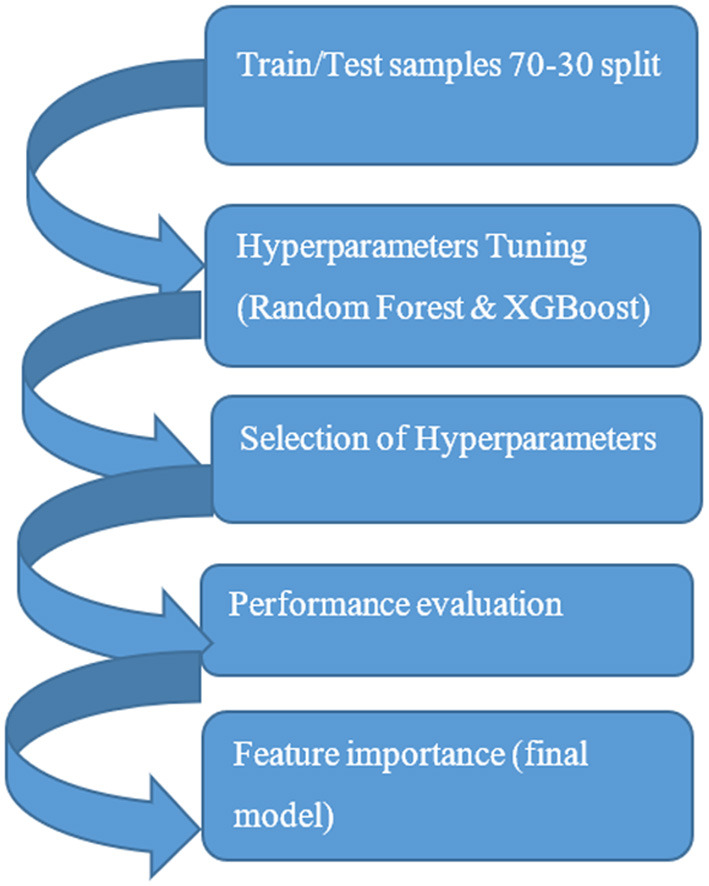
Predictive modeling workflow.

The objective for this study was to include predictive power in the data framework. Preventable adverse event was flagged when medication from EHR data was part of the CPIC level A (sufficient evidence for at least one prescribing action) grouping and contraindicated with the prototyped genetic data allele as defined by CPIC guidelines. Predictive Analytics using machine learning was used as a methodology to assess the predictive power of the model as part of the larger integrated data framework. The decision support prototype compared five machine learning methods and compared the output for accuracy. A common supervised learning workflow was applied to building the predictive models in the study. For the machine learning models, the first step was training and testing partitioning, 70 and 30 percent samples. Hyperparameters tuning for the models using k-fold cross validation was performed with selection of the best three hyperparameters for each of the three final prediction models. Figure 2 is a depiction of the predictive modeling workflow.

For the machine learning models, after data was split into training (70%) and the testing sets (30%), stratified sampling was used to ensure that proportion of classes are the same in the training and testing sets. To be able to perform hyperparameters tuning, k-fold cross validation was used. In V-fold cross validation a resampling method is used in which data are randomly partitioned into V folds of approximately equal size. In literature, a well reputed hyperparameters tuning is 10-fold cross validation which gives an idea of true out of sample performance. Some methods such as bootstrapping may be biased. As part of the data analysis, the outcome variable for predicting an adverse event based on the person's medication and prototyped contraindicating gene allele data was evaluated for performance of the algorithm over clinical relevance of the results.

The hyperparameters related to the types of models and their descriptions used for this study are tabulated in Table 2.

**Table 2:** Hyperparameters used in models.

**Model**	**Hyperparameter**
Random Forest	max_depth: longest path between root node and leaf node n_estimators: Number of trees in the forest = 10 criterion: [“entropy', 'gini”] = “gini” max_features: Number of maximum features for each tree = “log2” min_samples_split: Minimum number of observations in any given node in order to split it = 5 min_samples_leaf: Number of samples that should be present in the leaf node after splitting a node = 1 n_estimators: Number of trees = 200
XGBoost	colsample_bytree: Percentage of features for building each tree = 0.55 learning_rate: Gain with each iteration = 0.1 n_estimators: Number of trees = 200 reg_lambda: parameter to apply regularization = 100
SVM	C: “adds a penalty for each misclassified data point. If c is small, the penalty for misclassified points is low so a decision boundary with a large margin is chosen at the expense of a greater number of misclassifications (0.1 < c < 100)” = 1000 gamma: degree of similarity or closeness (0.0001 < gamma < 10) = 0.0001 kernel = rbf
Decision Tree	max_features: Number of maximum features for each tree = “log2” n_estimators: Number of trees in the forest criterion': [“entropy”, “gini”] = “entropy” max_depth': Number of maximum features for each tree = 50 min_samples_split: Minimum number of observations in any given node in order to split it = 2 min_samples_leaf: Number of samples that should be present in the leaf node after splitting a node = 5
KNN	n_neighbors: Number of neighbors = 5 weights: uniform or distance = “uniform” algorithm: specific algorithms type = “brute” leaf_size: maximum points a node can hold = 10

As part of the tuning process performance metrics were obtained for each set of the hyperparameters. Based on 10-fold cross validation, the best model was used to get predictions on the testing sample and final performance metrics were reproduced. For regression models, root mean square deviation (RMSE) metric was used as performance measure. Area under curve (AUC) is used for evaluating regression models with receiver operating characteristic (ROC) analysis.

Given the imbalanced distribution of the outcome variable the weight of each class of the outcome variable was balanced before training each model. For each model, grid search technique was utilized to find the optimal hyperparameters to get the best performance. The prediction models were conducted with five different methods. For the first step, total medications, HIV, diabetes, cardiovascular disease, any cancer, and number of diseases were included. For the second step, the Gene Allele variable was added to the first model to analyze if genetic data improved the prediction of PAE. For the first step five different machine learning methods (KNN, Random Forest, SVM, XGBoost and Decision Tree) were compared to finalize the type of final prediction model. For the first step with no genetic data, all AUC scores on training and testing datasets across models were compared. For additional validation, an XGBoost model without the gene predictor was created to evaluate if the prediction of PAE improved with presence of genetic data. AUC scores were compared between the final models before and after adding gene in the testing dataset.

## Results

Amongst the five models, XGBoost performed most efficiently as the final model to predict PAE. Random Forest, Decision Tree and KNN had slight overfitting issues when comparing AUC between training and testing datasets. Decision Tree and KNN had the lowest AUC indicating relatively weaker performance. The results of modeling helped evaluate feasibility of the framework and explore performance of the five given methods.

Table 3 shows the ROC-AUC scores obtained for all models for the training and testing datasets. On the testing datasets, AUC scores were the highest for SVM and XGBoost.

**Table 3:** ROC-AUC score comparison.

		**Random forest**	**XGBoost**	**SVM**	**Decision tree**	**KNN**	**Ensembling**
ROC-AUC	Training dataset	0.980	0.975	0.968	0.973	0.975	0.980
	Testing dataset	0.969	0.972	0.975	0.960	0.957	0.969

Though two relatively better performing models, XGBoost and SVM had high performance XGBoost had higher F1 score than SVM especially for minority observations. Classification or categorizing of data was based on absence of gene (given a value of 0) or presence of gene (given a value of 1) for the person's given medication as defined by CPIC (CPIC, 2021). This indicated a better balance of precision and recall for the XGBoost model shown in Table 4.

**Table 4:** Classification report for XGBoost and SVM.

**Classification**	**Precision**	**Recall**	**F1-score**	**Support**
**XGBoost**				
0	0.55	0.97	0.70	78
1	1.00	0.91	0.95	664
**SVM**				
0	0.50	1.00	0.67	78
1	1.00	0.88	0.94	664

**Figure 3:**
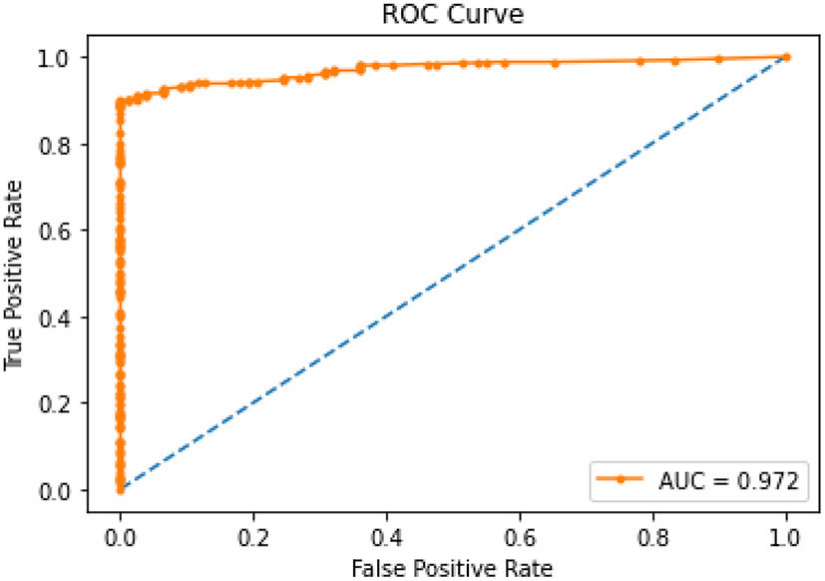
ROC Curve for XGBoost model.

Figure 3 illustrates the ROC curves for the XGBoost model that showed an optimal threshold of 0.319.

**Figure 4:**
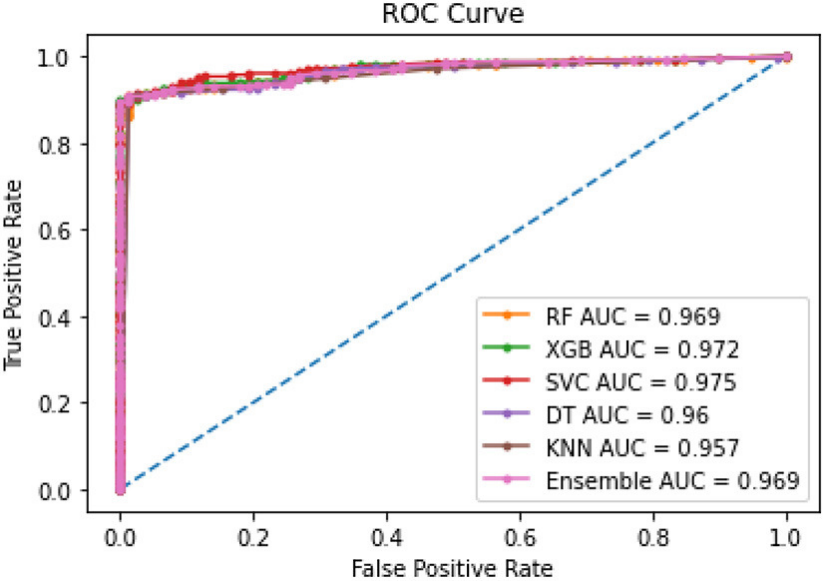
ROC for all model comparison.

The combined ROC curves for the five models shown in Figure 4 are almost overlapping with each other indicating all model performances were in the acceptable range.

Once presence of gene contraindicated with the medication was added to the XGBoost model it showed higher AUC scores (0.972) as compared to the first model without the gene variable (0.766). Table 5 shows the area under the curve scores for the XGBoost model.

**Table 5:** ROC-AUC scores for XGBoost.

	**XGBoost**	
	**Training dataset**	**Testing dataset**
Model 1	0.746	0.766
Model 2	0.975	0.972

AUC scores were also compared on test data before and after applying SMOTE. Results indicated that resampling did not impact the model. The full results of the analysis can be found with the supplementary material accompanying this paper.

Therefore, we can conclude that adding genetic variable (presence/absence of gene contraindicating with person's medication) into the model improved prediction of PAE. The optimal threshold probability was obtained by using ROC scores and maximizing the sum of sensitivity and specificity. We got an optimal cut off probability of 0.319. The ROC-AUC score on the training dataset was 0.975 and the testing dataset was 0.972. The recall and sensitivity were 0.920 which means 92% of PAE's were identified by this model. The precision was 0.990 which means, of all PAE's identified by the model 99% were true cases. The F1 score was 0.960. The predictors based on their importance were gene, number of total medications, diabetes, HIV followed by number of conditions, cancer, and cardiovascular disease. Therefore, we can conclude that adding genetic variable into the model improved prediction. For the final model, the F1 score without the gene variable was 0.73 with a precision of 0.59 and a recall of 0.94. After gene was added to the model, the F1-score was 0.96 with a precision of 0.99 and recall of 0.92 (Table 6). The classification or categorization of labels is based on presence (value of 1) or absence (value of 0) of contraindicated gene for the person's medication based on CPIC guidelines.

**Table 6:** Classification report for final model.

**Classification**	**Precision**	**Recall**	**F1-score**	**Support**
0	0.59	0.94	0.73	78
1	0.99	0.92	0.96	664

**Figure 5:**
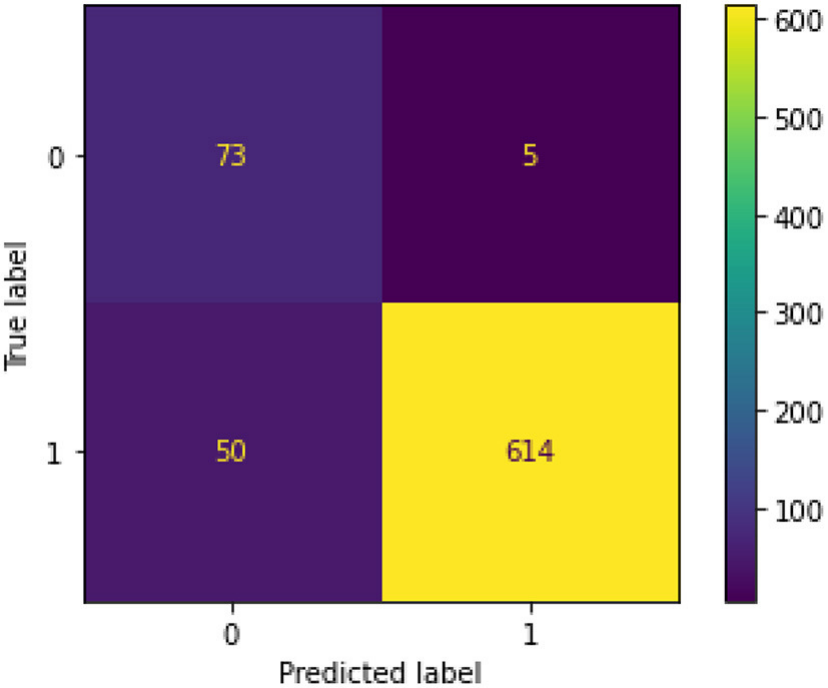
Confusion matrix for final XGBoost model.

Figure 5 illustrates the confusion matrix for the final XGBoost model.

**Figure 6:**
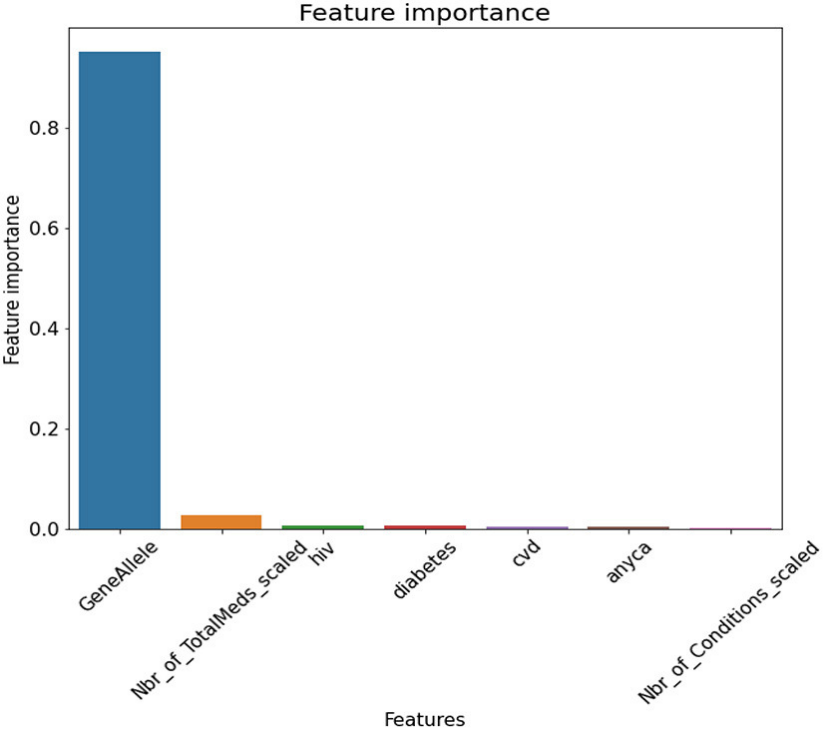
Feature Selection for Final Model. GeneAllele = Variable for presence/absence of gene contraindicated with medication, Nbr_of_TotalMeds_scaled = scaled variable for number of total medications, hiv = presence/absence of hiv, diabetes = presence/absence of diabetes, cvd = presence/absence of cardiovascular disease, anyca = presence/absence of cancer, Nbr_of_Conditions_scaled = number of total disease conditions.

Feature selection showed in order of importance variables, gene allele followed by number of medications a person was on. The conditions seemed to be close to each other in feature importance. Figure 6 depicts the feature selection for the final model.

**Figure 7:**
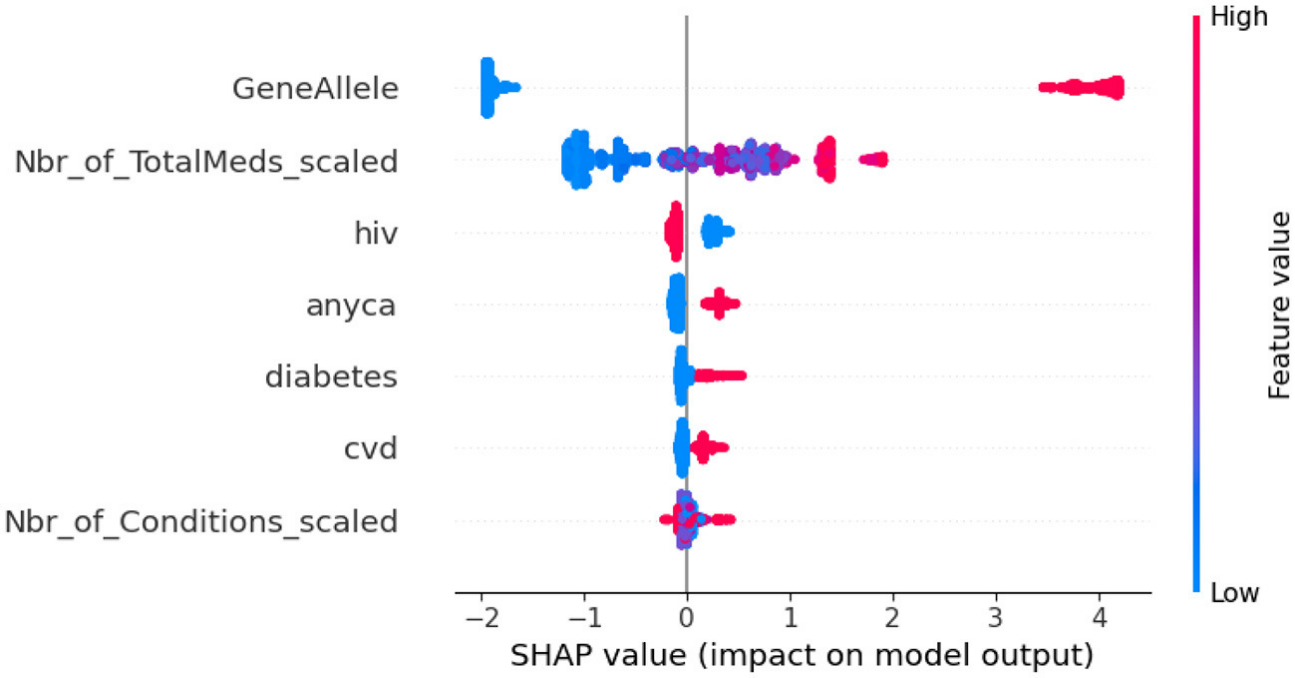
SHAP values for XGBoost model. GeneAllele = Variable for presence/absence of gene contraindicated with medication, Nbr_of_TotalMeds_scaled = scaled variable for number of total medications, hiv = presence/absence of hiv, diabetes = presence/absence of diabetes, cvd = presence/absence of cardiovascular disease, anyca = presence/absence of cancer, Nbr_of_Conditions_scaled = number of total disease conditions.

To do additional exploration between variables, bivariate chi-square tests were performed between dependent variables and categorical independent variables. Results indicated PAE was significantly related to “GeneAllele” (variable for presence/absence of gene contraindicated with medication) with a *p*-value < 0.001. For explaining the impact of each feature variable on the target variable PAE, Shapely Additive Explanations (SHAP) was utilized on the final XGBoost model. SHAP values were generated using TreeExplainer. A beeswarm plot was generated to visualize the global feature importance. The plot in Figure 7 shows “GeneAllele” (variable for presence/absence of gene contraindicated with medication) as the topmost important feature that impacts prediction of PAE.

## Discussion

### Reflections on an integrated framework

Most people access healthcare at some point in their lives. Healthcare utilization typically increases with aging, and its operational efficiency directly affects quality of life and wellbeing (Hallo and Gorod, 2020). Therefore, it is of utmost importance that we apply expertise of different disciplines to enhance the efficiency and output of the healthcare industry. Previous work has shown that innovation is the least studied category in health-related operations research (Brownlee and Garber, 2019). Application of methods like predictive analytics that have been successfully used in other industries have been slow in being adopted in healthcare. One main reason for this is the complexity of the healthcare system. Predictive modeling and comparison of different types of models has not been applied to clinical decision making in the pharmacogenetic domain in outpatient care at the VA. This research is one step in the attempt to apply these methods to improve patient care efficiency.

The VA serves more than 13 million veterans with more than 1600 points-of-care nationwide. Routine patient care does not typically incorporate factoring genetic data to make decision strategies (Dong et al., 2021). Several institutional initiatives are looking to incorporate genetic data (Zhang, 2016), but they are generally restricted to specific sites. Previous studies show that if patient care is personalized based on the genetic constitution of the person, then outcomes are more successful. Fewer resources are spent in addressing adverse reactions and additional care brought on by the least optimal decision. A data framework integrated with genetic data decision support aided with machine learning methods can aid patient care decisions guided by pharmacogenetics. In many healthcare systems around the world, even with best human clinical expertise the outcome of treatments is not always satisfactory, whereas systemically when operational efficiency of healthcare systems is high, treatment outcomes are much better (Tolk et al., 2015). Therefore, based on this study's evaluation we propose wholesome data frameworks that systemically support informed clinical decision making.

## Conclusion

Tree-based ensemble ML models closely mirror a human's decision making and have better explainable-ability and predictive performance along with being robust to outliers. For this study five different machine learning algorithms were trained to explore better performing models for predicting outcomes. The model performance on all the machine learning models was acceptable based on the ROC-AUC scores. XGBoost performed relatively better but all model predictions were acceptable, indicating that the methodology may be an effective way to utilize the power of machine learning for predicting PAE in an integrated data framework.

## Data availability statement

The datasets presented in this article are not readily available because due to security regulations of the Department of Veterans Affairs a link cannot be provided outside of the VA firewall. Requests to access the datasets should be directed to the corresponding author.

## Ethics statement

The studies involving human participants were reviewed and approved by the Veterans Aging and Cohort Study, Department of Veterans Affairs. The patients/participants provided their written informed consent to participate in this study.

## Author contributions

FK-K was responsible for concept, design, analysis, and manuscript preparation. CB and AJ were responsible for manuscript editing and supervision. All authors contributed to the article and approved the submitted version.

## Funding

This work was supported by the National Institute on Alcohol Abuse and Alcoholism grants: U01-AA026224, U24-AA020794, U10 AA013566, and U01 AA020790.

## Conflict of interest

The authors declare that the research was conducted in the absence of any commercial or financial relationships that could be construed as a potential conflict of interest.

## Publisher's note

All claims expressed in this article are solely those of the authors and do not necessarily represent those of their affiliated organizations, or those of the publisher, the editors and the reviewers. Any product that may be evaluated in this article, or claim that may be made by its manufacturer, is not guaranteed or endorsed by the publisher.

## Author disclaimer

The content is solely the responsibility of the authors and does not necessarily represent the official views of the Veterans Affairs or the National Institutes of Health.
